# Optimizing DUS testing for *Chimonanthus praecox* using feature selection based on a genetic algorithm

**DOI:** 10.3389/fpls.2023.1328603

**Published:** 2024-01-18

**Authors:** Ting Zhu, Yaoyao Feng, Xiaoxuan Dong, Ximeng Yang, Bin Liu, Puying Yuan, Xingrong Song, Shanxiong Chen, Shunzhao Sui

**Affiliations:** ^1^ Chongqing Engineering Research Center for Floriculture, Key Laboratory of Agricultural Biosafety and Green Production of Upper Yangtze River (Ministry of Education), College of Horticulture and Landscape Architecture, Southwest University, Chongqing, China; ^2^ College of Computer and Information Science, Southwest University, Chongqing, China; ^3^ Garden and Flower Research Center, Horticultural Research Institute of Sichuan Academy of Agricultural Science, Chengdu, China

**Keywords:** wintersweet, DUS test, feature selection, genetic algorithm, core feature

## Abstract

*Chimonanthus praecox* is a famous traditional flower in China with high ornamental value. It has numerous varieties, yet its classification is highly disorganized. The distinctness, uniformity, and stability (DUS) test enables the classification and nomenclature of various species; thus, it can be used to classify the *Chimonanthus* varieties. In this study, flower traits were quantified using an automatic system based on pattern recognition instead of traditional manual measurement to improve the efficiency of DUS testing. A total of 42 features were quantified, including 28 features in the DUS guidelines and 14 new features proposed in this study. Eight algorithms were used to classify wintersweet, and the random forest (RF) algorithm performed the best when all features were used. The classification accuracy of the outer perianth was the highest when the features of the different parts were used for classification. A genetic algorithm was used as the feature selection algorithm to select a set of 22 reduced core features and improve the accuracy and efficiency of the classification. Using the core feature set, the classification accuracy of the RF model improved to 99.13%. Finally, K-means was used to construct a pedigree cluster tree of 23 varieties of wintersweet; evidently, wintersweet was clustered into a single class, which can be the basis for further study of genetic relationships among varieties. This study provides a novel method for DUS detection, variety identification, and pedigree analysis.

## 1 Introduction

*Chimonanthus praecox* (wintersweet) is an ornamental tree commonly found in China. Wintersweet, an excellent and rare winter ornamental garden plant with high garden application value, blooms in the winter with a golden color and emits a pleasant scent. Owing to its cultural significance and long-standing history, it boasts a wide variety of cultivations. However, owing to the lack of clear classification norms and systems, proper communication and consensus regarding the statistics on the number of wintersweet varieties are lacking. Consequently, researchers use their own schools, which has resulted in “synonyms” and “foreign bodies of the same name.” This has resulted in some serious problems, such as the exact number of its varieties and their names being unknown (Chen and Lu, 2001). This not only affects the actual production of varieties but also the statistics, classification, and breeding of wintersweet resources, thus limiting the development of wintersweet seedlings. In 2013, China formulated and issued the “Guidelines for the conduct of test for distinctness, uniformity and stability—Wintersweet [*Chimonanthus praecox* (L.) Link.].” The application of new varieties must be reviewed and tested by the distinctness, uniformity, and stability (DUS) test to ensure that the new varieties are protected by law. The DUS test has played a key role in resolving confusion in the identification, management, and naming of wintersweet varieties. To date, only 27 varieties of wintersweet have been internationally registered (Chen et al., 2017; Chen et al., 2020), which is far below the statistics reported by Chinese researchers (Lu et al., 2010; Lu and Li, 2011; Lu and Ren, 2011; Lu et al., 2012; Song et al., 2012). The small number of registered varieties is not conducive to the legal spread and trade of wintersweet varieties worldwide. Therefore, establishing an effective identification system for new plant varieties is crucial for variety management and intellectual property rights (Fister et al., 2017).

DUS testing is a complex process based on the study of plant morphological characteristics (Bernet et al., 2003). At present, DUS testing of new plant varieties is carried out manually; however, owing to the heavy workload of evaluation, the possibility of errors is increased in the process of measurement and evaluation. Additionally, the evaluation results are often inconsistent because different testers have different subjective perceptions of characteristics; moreover, the strategy of employing manual testers has the limitations of yielding low efficiency and incurring high labor costs (Kwon et al., 2005; He et al., 2020). In theory, as the number of tested traits increases in a DUS, the recognition rate improves accordingly. However, in actual classification, information redundancy occurs in DUS, thus leading to a decrease in recognition efficiency (Deng and Han, 2019). For example, the peanut DUS test comprises a total of 37 important candidate features, and when only 18 of these features are used, the recognition rate exceeds 90% (Deng and Han, 2019). In a previous study, among the 50 basic DUS traits specified for cucumber plants, 20 core traits were evaluated; consequently, the efficiency of variety identification improved significantly, and up to 60% of labor and time costs were saved (Zhang et al., 2022). Therefore, scientific trait combinations can be screened to efficiently identify varieties.

With continuous developments in computer science, researchers have applied pattern recognition and machine learning techniques to plant recognition (Sachar and Kumar, 2021), disease detection (Sharif et al., 2018), and plant genotype prediction (Heidari et al., 2020). This has led to the rapid development of automation in the field of plant operation and compensated for the limitations of low efficiency and high cost of manual work. Pattern recognition, an automatic intelligent technology that is similar to biochemical and molecular technologies, has become widely popular for plant DUS testing and has been recognized by the International Union for Conservation (Deng and Han, 2019). Zhao et al. (2009) used the pattern recognition correlation method to quantify DUS test traits in corn ears. This method has the advantages of objectivity, high efficiency, and low cost. Moreover, they believed that relevant technology will play an increasingly important role in DUS testing of other new plant varieties. She et al. (2019) proposed an improved least squares support vector machine pixel classification method to classify lawn plants. Nijalingappa and Madhumathi (2015) used a multiclass support vector machine as a classifier and proposed a plant recognition method based on leaf features, which can effectively realize plant classification. Using a multiclass support vector machine as a classifier, Nijalingappa and Madhumathi (2015) proposed a plant recognition method based on leaf characteristics, which can effectively realize plant classification. Computer technology can replace manual and automatic plant morphology measurements. It has various advantages, such as strong recognition ability, short processing time, and good repeatability and objectivity (Donis-Gonzalez et al., 2013); additionally, it can compensate for the shortcomings of traditional DUS testing methods (Deng and Han, 2019).

When establishing efficient classification models for plant recognition, the information contained in the features should be as rich as possible, and the number of features should be as small as possible, which makes feature selection a crucial step. Inappropriate features may lead to a sharp increase in the number of data dimensions, the introduction of noise features, or an unnecessary increase in complexity, which may weaken the performance and generalization ability of the model. Redundant or irrelevant features not only increase the computational cost but may also lead to a reduction in classification accuracy (Ghasab et al., 2015; Khan et al., 2019). Therefore, the best discriminant features must be determined for an efficient classification. In the identification of apple diseases, Khan et al. (2019) optimized the extracted features using a genetic algorithm (GA) and a multi-support vector machine for classification. The results showed that this method could improve the classification accuracy for apple diseases. Kheirkhah and Asghari (2019) adopted principal component analysis (PCA) to select the best features from a plant leaf feature set, which improved the recognition accuracy and speed. Elnemr (2017) extracted and normalized leaf texture features; subsequently, they employed the neighborhood component feature selection method to reduce the feature space and selected 16 important features that can effectively distinguish different categories. Consequently, the accuracy of the k-nearest neighbor (kNN) algorithm reached 98%. Ghasab et al. (2015) used the ant colony algorithm to search the internal feature search space to obtain the best feature set for leaf recognition and then used a support vector machine (SVM) to classify species with an accuracy of 95.53%. For the rapid classification and identification of honeysuckle leaf defects, Xiao and Wang (2020) employed PCA for feature extraction and selection, thereby achieving high accuracy and significantly improved recognition speed.

**Figure 1:**
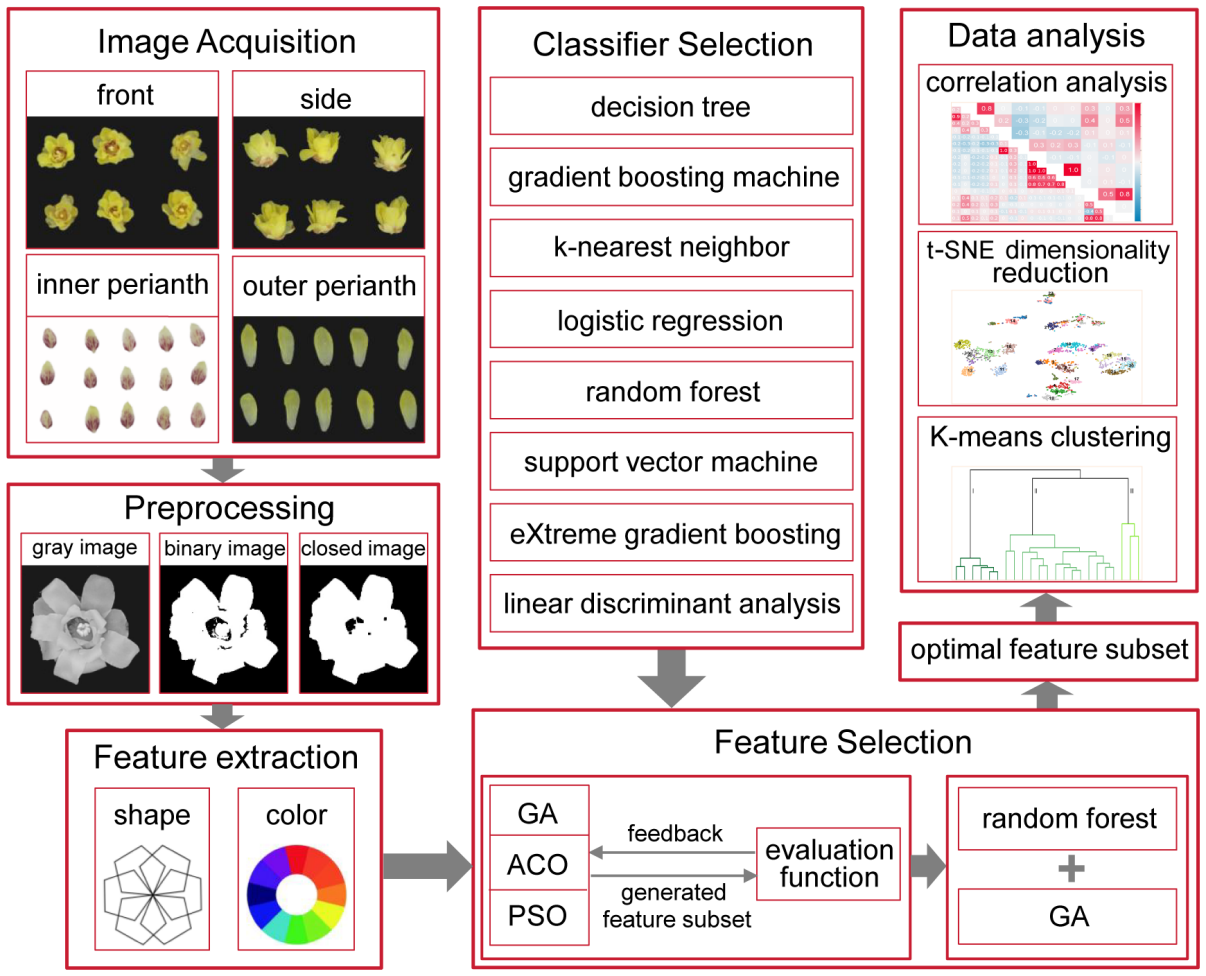
Research framework.

In plant recognition, different features, such as shape, color, morphology, texture, and vein structure, are extracted from images of leaves, fruits, flowers, and other plant organs to evaluate their impact on plant recognition (Ghasab et al., 2015). Vegetative organs are easily affected by the environment, and the characteristics of the reproductive organs are relatively stable (Li, 2009). Therefore, flower and fruit characteristics are crucial for plant recognition. In the classification of wintersweet, the leaf and fruit traits of different varieties vary very little, which contributes little value to the classification of wintersweet varieties (Zhao et al., 2007). In the DUS test of wintersweet, the number of flower traits tested corresponds to nearly two-thirds of all the traits. In previous studies, the classification of wintersweet varieties was aimed primarily at flowers; therefore, this study used floral organs as the research object. To optimize the accuracy of the DUS test of wintersweet and ensure the interpretability of the optimization process, a traditional machine learning method was used to accurately screen the traits in the test. The research framework of this study is shown in **Figure 1**. First, the flower images were preprocessed, the traits in the DUS test were quantified, and new features were proposed. Using these features, different algorithms were applied to classify varieties, and an optimal classification model was selected. Moreover, the importance of different parts of the flowers for variety classification was evaluated. Furthermore, all the features were used to form the feature search space, and a GA was used for feature screening to determine the core feature set of the wintersweet variety classification. The core feature set was used to classify the varieties, and the performance was compared with that of all the feature sets in the model to test the optimization effect. The distribution of features in low-dimensional space can be directly observed via t-SNE visualization. In addition, the k-means algorithm was used to analyze the potential genetic relationships among the varieties, which provided insight into their genetic background.

## 2 Materials and methods

### 2.1 Wintersweet sample and image acquisition

**Figure 2:**
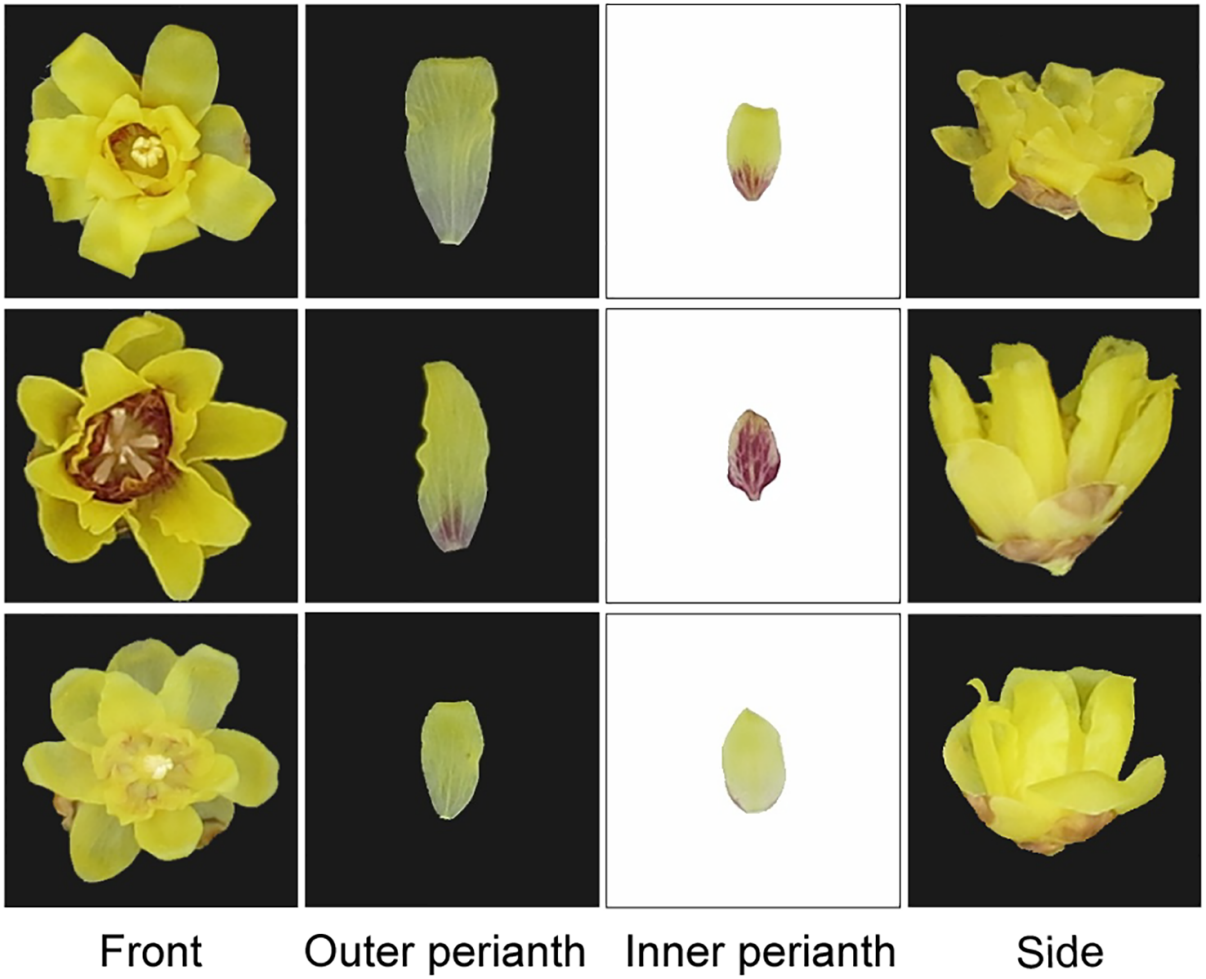
Front, outer perianth, inner perianth, and side views of three sample varieties (each line represents one variety).

Herein, 23 different wintersweet varieties were collected from the resource nursery, and 100 flowers in the bloom period were selected for each variety. The collected flowers were placed in a laboratory studio and photographed using a CanonPowerShotG1X camera. With respect to the camera parameters, the exposure times for the black and white backgrounds were 1/30 and 1/125 s, respectively, whereas the other parameters were consistent. The front, outer perianth, inner perianth, and sides of the flower were photographed (**Figure 2**), and 100 images were collected for each part, resulting in a total of 9,200 images.

### 2.2 Image preprocessing and feature extraction

**Figure 3:**
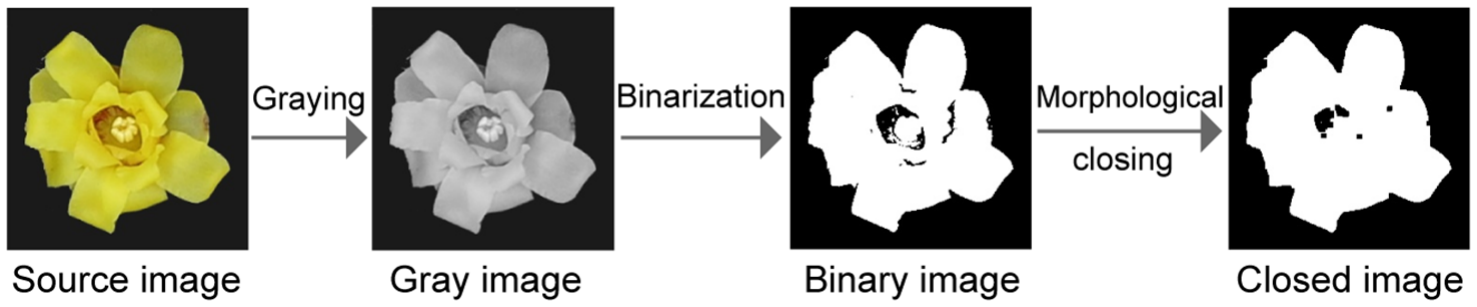
Schematic of the image preprocessing steps.

Image preprocessing is a key step in the initial stage of image analysis. Its purpose is to eliminate noise, enhance the important features of the image, and ensure the accuracy and reliability of the subsequent analysis. **Figure 3** illustrates the preprocessing steps used in this study. First, the collected images were converted into grayscale images, which can better highlight the contour and morphological information and reduce the complexity of the data processing. Next, binary processing was performed to highlight the structural characteristics of the wintersweet more clearly. In addition, a closed operation was employed to eliminate potential small noise or breaks. Specifically, the small noise was corroded first, and the main structure of the image was restored by the expansion step. Subsequently, the image contour was calculated to obtain its area, perimeter, and other key parameters, which provided the basis for subsequent classification and analysis. Images were transferred from the RGB color space to the HSV color space to capture the color characteristics of wintersweet more accurately. In the HSV color space, the pixel mean of each channel was calculated, which provides important information regarding the color for image analysis.

Based on the different types of preprocessed images, 30 features in the DUS test were quantified, and 12 new features were proposed. According to the parts, the features were divided into three categories: description of the overall (including shape and size), outer perianth, and inner perianth, totaling 42 features (**Table 1**).

**Table 1:** Characteristic list of the wintersweet blossom.

Feature ID	Feature of overall	Feature ID	Feature of outer perianth	Feature ID	Feature of inner perianth
F1	Flower type	F11	Number of outer perianth	F27	Number of inner perianth
F2	Maximum diameter	F12	Minimum rectangular length of outer perianth	F28	Minimum rectangular length of inner perianth
F3*	Perimeter	F13	Minimum rectangular width of outer perianth	F29*	Minimum rectangular width of inner perianth
F4*	Area	F14	Outer perianth shape	F30	Inner perianth shape
F5*	Equal circle area	F15*	Outer perianth area	F31*	Inner perianth area
F6*	Concave-convex ratio	F16	Outer perianth apex shape	F32	Inner perianth apex shape
F7*	Circularity	F17	Outer perianth apex state	F33	Inner perianth apex state
F8*	Compactness	F18	Outer perianth marginal state	F34	Average R value of inner perianth main color
F9	Outer perianth position relationship	F19	Average R value of outer perianth main color	F35	Average G value of inner perianth main color
F10	Outer perianth extension angle	F20	Average G value of outer perianth main color	F36	Average B value of inner perianth main color
		F21	Average B value of outer perianth main color	F37	Average R value of inner perianth secondary color
		F22*	Average R value of outer perianth secondary color	F38	Average G value of inner perianth secondary color
		F23*	Average G value of outer perianth secondary color	F39	Average B value of inner perianth secondary color
		F24*	Average B value of outer perianth secondary color	F40	Percentage of secondary color area of inner perianth
		F25*	Percentage of secondary color area of outer perianth	F41	Whether the inner perianth is polychromatic
		F26*	Whether the outer perianth is polychromatic	F42	Inner perianth secondary color distribution type

The features marked by * are those newly proposed in this study.

### 2.3 Application of classification algorithms

Various classification algorithms were selected to ensure the accuracy and robustness of the wintersweet classification, and their classification performances were compared. Among them, the decision tree (DT) (Myles et al., 2004) is suitable for the intuitive interpretation of the classification rules of wintersweet because of its clear decision path. In addition, gradient boosting machine (GBM) (Natekin and Knoll, 2013) and eXtreme gradient boosting (XGBoost) (Chen and Guestrin, 2016) methods provide advantages in dealing with complex data structures to improve classification accuracy. kNN (Guo et al., 2003) provides an instance-based learning method and another solution strategy for complex patterns in datasets. Logistic regression (LR) (LaValley, 2008) and SVM (Noble, 2006) are considered for their ability to address linearly separable and nonlinear problems. Linear discriminant analysis (LDA) (Wei and Croft, 2006) can reduce the dimensionality of the data while ensuring the maximum distance between categories; thus, this approach is helpful for subsequent data visualization and classification. RF (Statistics and Breiman, 2001) provides high accuracy and adapts to high-dimensional data by combining the prediction results of multiple decision trees; thus, it was also applied to our classification problems.

The constructed dataset was divided into training and test sets at a ratio of 0.75:0.25 to ensure the stability of the training and accuracy of the evaluation. In addition, during the experiment, five-fold cross-validation was performed for each classifier to obtain a more stable performance evaluation. The performances of these eight algorithms were comprehensively compared, and the model with the best performance was selected as the basis for subsequent feature selection and further optimization.

### 2.4 Feature selection procedure

The importance of feature selection has been widely recognized (Li et al., 2018). The high number of features extracted from the wintersweet image (42 features) ensured feature diversity; however, this high number presented the dilemma of high dimensionality, which may affect the classification. Therefore, feature selection was necessary. The feature set obtained after screening is not only more concise and intuitive but also helps improve the interpretability of the model (Piri and Mohapatra, 2021), thus providing a theoretical basis for further research on the characteristics related to the recognition of wintersweet varieties.

Common feature selection methods include filter, wrapper, and embedded methods (Saeys et al., 2007). The wrapper feature selection method comprehensively considers the interactions and nonlinear relationships among features and reflects the quality of the feature set based on the performance of the current feature set in the model to ensure that the feature set is more consistent with the model. Therefore, the wrapper method was used for feature selection in this study.

To select the most suitable feature subset in the feature selection process, the following steps are usually used: First, a feature subset is generated from the initial feature set, and the next subset is generated by evaluating its advantages and disadvantages. Then, the newly generated subset is evaluated, and the next subset is generated according to the evaluation results. This cycle is iterated until no better feature subset can be found. Therefore, in the process of feature selection, the search and evaluation of feature subsets are the most critical, and they have an important effect on the quality of the final selected feature subset (Dash and Liu, 1997).

Therefore, in the wrapping method, the best performing classifier is encapsulated with the search algorithm, and the evaluation function is embedded as a criterion to measure the quality of the selected feature set.

#### 2.4.1 Search for feature subsets

The feature subset search algorithm is an important tool for optimizing feature selection and can significantly improve the performance of machine learning models. The ant colony algorithm (ACO) (Dorigo et al., 2006), GA (Holland, 1992), and particle swarm optimization algorithm (PSO) (Poli et al., 2007) are widely used in feature subset searches and have stable performance and good feature reduction ability. These methods are combined with classifiers to find excellent feature subsets. In this study, the ACO, PSO, and GA algorithms were tested, and based on their performance, the GA (an evolutionary-inspired global optimization algorithm) was selected. The GA considers the interaction between features and adaptively searches for the optimal feature subset. Thus, the solution closest to the optimal solution is determined, which has a certain robustness and global search ability (Smith et al., 2012).

#### 2.4.2 Evaluation function

The evaluation function plays a key role in feature selection, as it directly affects the quality of the final selected feature subset (Dash and Liu, 1997). In this study, the evaluation function was embedded in the wrapper method; that is, the feature sets of different combinations were evaluated by the classifier, the best feature sets were updated over time, and the final feature sets were obtained after several iterations. In the wrapping process, the evaluation of the feature subset is based on the performance of the current feature subset on the target classifier (i.e., it is evaluated by combining the current error rate and current number of features), and the performance must simultaneously meet the requirements of a low error rate and small number of features to improve the overall performance and interpretability of the model. Therefore, the evaluation function of this study is expressed as follows:


Cost(St)=λ1E(St)+λ2N(St)

where t is the number of the current iteration, St is the feature subset selected in the iteration process, E(St) is the error rate of using the feature subset on the classifier, N(St) is the length of the feature subset, and λ 1 and λ 2 are adjustable parameters that are used to regulate the weight of the error rate and the selected number of features in the evaluation function, respectively. The ratio of the mean error rate to the mean number of features is used to determine the order of magnitude gap between the two, and based on this, appropriate fine-tuning is used to determine the final values ​​of λ1 and λ2, ranging from [0,1]. This implies that the subset selected in this study was the global optimal subset that satisfies both the minimum error rate and the shortest feature subset length.

#### 2.4.3 Search termination criteria

The last criterion that must be clearly stated in the algorithm is the search process termination condition. The objective of the stop condition is to provide accurate guidance for the feature search process, essentially avoiding infinite loops or excessive searching. In this study, the stop condition was used to achieve the maximum number of iterations and minimize the value of the evaluation function. This ensured that the feature search was completed in a limited time and that a relatively optimal feature subset was found.

#### 2.4.4 Structure of the GA

The GA is an optimization algorithm that simulates the process of natural evolution (Leardi et al., 1992). The fitness of individuals in a population is gradually improved through the basic steps of the GA. Through a series of iterations, the algorithm constantly updates the population via selection, crossover, and mutation operations to gradually approach the optimal solution.

**Figure 4:**
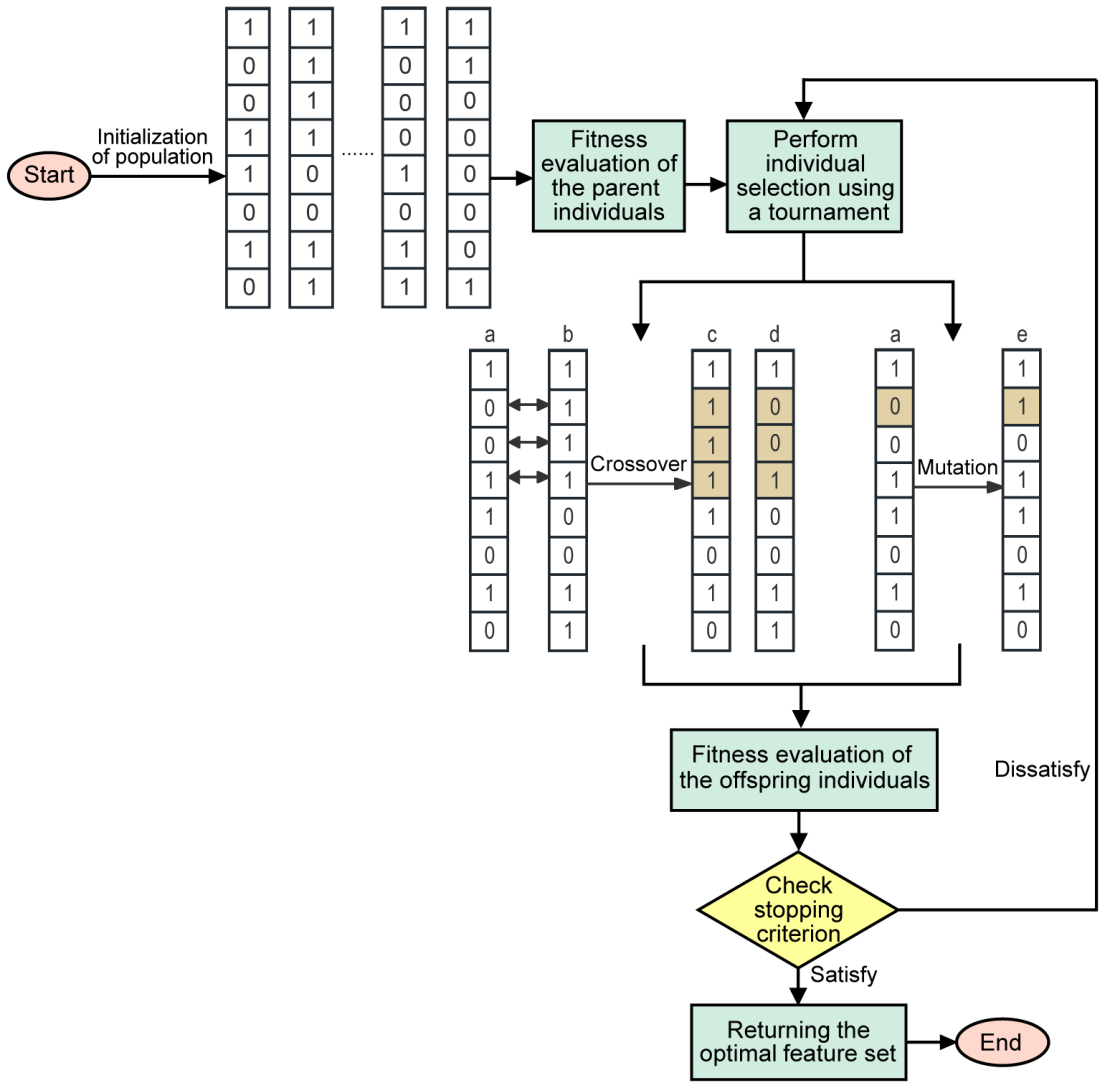
Flow chart representation of GA feature selection.

The workflow of the GA can be simplified as follows (**Figure 4**): First, initialization is performed to generate individuals with the same number of features. Each individual represents the selected status of the feature in binary code (1 represents selected, 0 represents unselected). Then, individuals are evaluated by a fitness function and selected by the tournament strategy. The selected winners undergo crossover and mutation operations to increase diversity and generate the next generation of individuals. Crossover operations involve exchanging genes to create new individuals, while mutation operations randomly change an individual’s genes. After the offspring individuals are reevaluated, if the stop condition is met, the algorithm returns the best feature subset. Otherwise, the tournament continues to select the winner, and the above steps are repeated until the stop condition is met.

Parameter selection of the GA is very important, and it is necessary to comprehensively consider the performance of the algorithm, convergence speed, final result, and rational utilization of resources. This study alternately optimized the parameters several times to select appropriate parameters. After many experiments, the final parameters were set as follows: the population size was 50 to ensure a wide search for the solution space, 20 generations were selected as the number of iterations to balance the computational cost and the quality of the solution, the tournament selection strategy was adopted, and the tournament size was three (i.e., three individuals were randomly selected for competition in each generation), and the one with the highest fitness was selected as the next generation parent individual.

## 3 Results

### 3.1 Classification abilities of different parts of the wintersweet

To identify the models with the strongest classification ability, eight commonly used machine learning models, namely, DT, GBM, kNN, LR, RF, SVM, XGBoost, and LDA, were used herein. Accuracy and F1-scores were used to evaluate the performance of the model. The experimental results showed that the RF model performed the best when all features were used for classification, with an accuracy of 98.81% (**Table 2**). When using only the 28 flower features of the DUS test, the RF model exhibited the highest classification accuracy (98.43%). This indicates that the traits proposed in the DUS test have good classification ability for wintersweets. When classified by parts, the SVM classifier was used to classify the outer perianth, and it achieved the highest accuracy (91.68%). The accuracy of the inner perianth was high (86.12%) when the XGBoost model was used. These results indicate that both the outer and inner perianths play crucial roles in the classification of wintersweet varieties. In particular, the outer perianth has the strongest classification ability; thus, this information can be used as an important basis for the classification of wintersweet varieties. Among the eight classification models, the RF and SVM models exhibited the best performance. The RF model had the highest accuracy when all features were used in the DUS test. Moreover, the RF model achieved the second highest accuracy for classifying the other three sets of features. This indicates that the RF model exhibits high classification accuracy.

**Table 2:** Classification results of 23 varieties by eight algorithms.

	All		DUS		Entirety		Outer perianth		Inner perianth	
	Acc	F1	Acc	F1	Acc	F1	Acc	F1	Acc	F1
DT	92.31%	92.23%	91.27%	91.03%	49.98%	49.65%	81.14%	81.02%	75.37%	75.42%
GBM	96.87%	96.85%	95.86%	95.79%	51.82%	52.04%	87.72%	87.35%	84.14%	84.58%
kNN	95.23%	95.06%	94.89%	94.77%	54.29%	53.91%	85.04%	84.69%	72.13%	71.77%
LR	98.12%	98.03%	96.35%	96.31%	53.11%	51.56%	88.80%	88.74%	79.26%	78.61%
RF	**98.81%**	**98.72%**	**98.43%**	**98.43%**	**55.05%**	**54.75%**	**90.92%**	**90.91%**	**85.28%**	**85.19%**
SVM	**98.22%**	**98.10%**	**97.81%**	**97.82%**	**56.83%**	**55.55%**	**91.68%**	**91.64%**	82.74%	82.61%
XGBoost	98.05%	98.05%	97.57%	97.55%	53.70%	53.32%	89.28%	89.12%	**86.12%**	**86.15%**
LDA	98.54%	98.53%	97.36%	97.31%	51.30%	50.25%	91.58%	91.61%	80.59%	80.67%

Bolded red font indicates the highest score, whereas only bold font indicates the top two with the highest scores.

### 3.2 Analysis of wintersweet feature characteristics and importance

**Figure 5:**
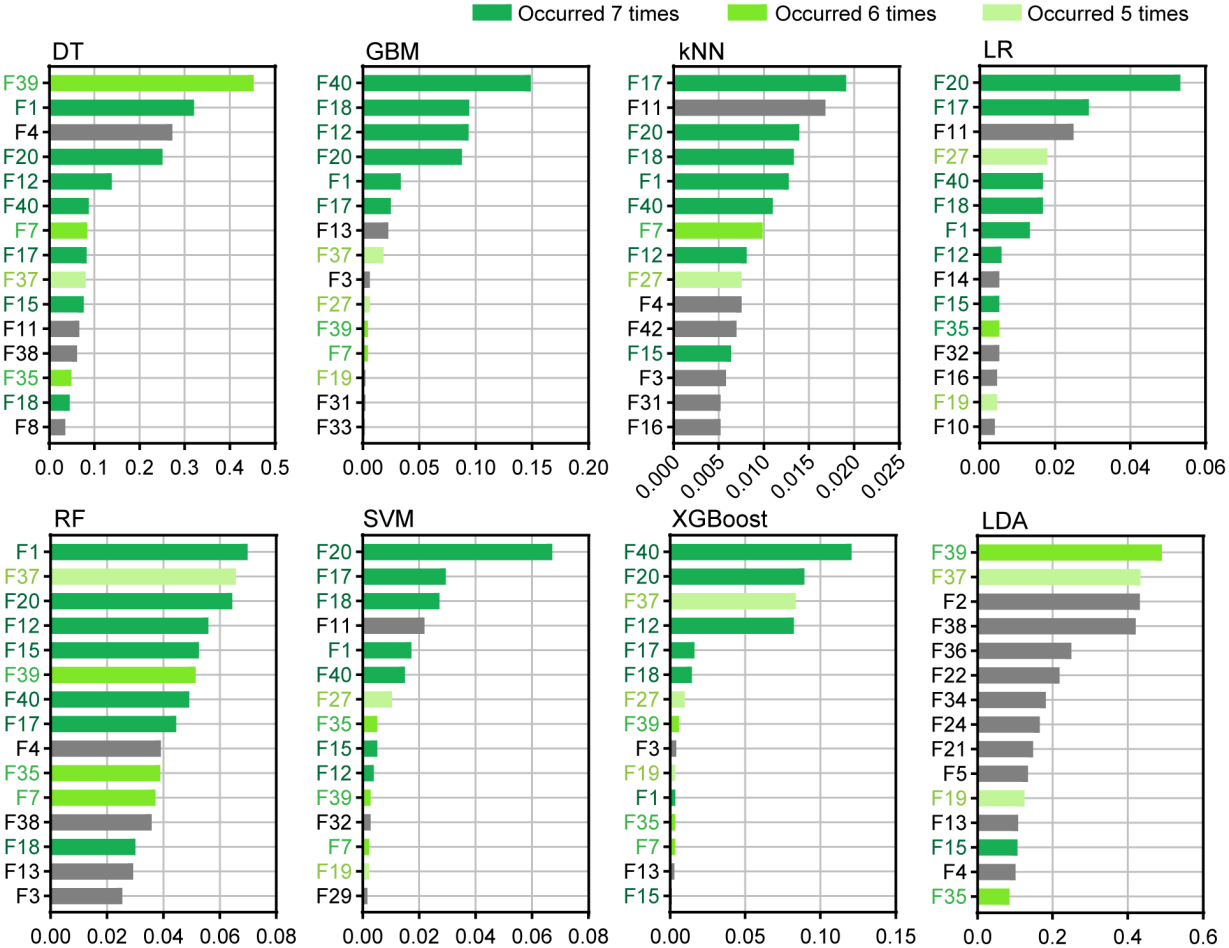
Top 15 important features of the eight algorithms.

The 15 most important features of each algorithm were ranked, as shown in **Figure 5**. The experimental results showed that for the DT and LDA algorithms, F39 was the most important feature. For GBM and XGBoost, F40 ranked first, and F39 and F40 were related to the color characteristics of the inner perianth. In the kNN model, F17 ranked first. For LR and SVM, F20 ranked first. F17 and F20 are related features of the outer perianth. In the RF model, F1 ranked first in importance. Among the first 15 features, features F1, F12, F15, F17, F18, F20, and F40 appeared in seven algorithms (dark green). Except for F1 and F40, all the features were associated with outer perianth-related traits. Additionally, F7, F35, and F39 appeared for six algorithms (green), whereas F19, F27, and F37 appeared for five algorithms (light green). Among the features, except for F7 and F19, the other features were associated with inner perianth traits. These features may play an important role in the classification of wintersweet.

### 3.3 Core feature selection

Although the highest recognition rate of varieties reached 98.81% when all 42 features were used, some redundancy may exist among the features owing to the excessive number of features considered. This redundancy can reduce the recognition efficiency and increase the difficulty in practical application. Therefore, the GA was used to screen features to improve classification accuracy and reduce implementation costs.

Herein, the wrapping method was employed to select features for RF model optimization because the overall performance of the RF model was better than that of the other models. The error rate of the variety classification and the length of the feature subset under the current iteration number were considered the two factors of the evaluation function. The results in **Table 3** suggest that among the five groups of better feature sets selected by each of the algorithms, GA, ACO and PSO, the third repetition of the GA corresponds to the smallest number of features and has the highest classification accuracy. When only 22 features were used, the accuracy of the model reached 99.13%, which is higher than the best accuracy obtained using all the features. The results showed that these 22 features play a key role in the classification task of wintersweets and can significantly improve the performance of the model. Therefore, the feature set of the third group was considered the core feature.

**Table 3:** Results of the feature selection based on GA, ACO, and PSO.

Algorithms	Repetition	Feature subsets	Length of subsets	Accuracy
GA	1	F1, F2, F4, F5, F7, F8, F11, F12, F14, F15, F9, F16, F17, F18, F19, F20, F21, F22, F23, F25, F27, F28, F30, F31, F35, F39, F40, F41, F42	29	99.13%
	2	F1, F3, F4, F6, F7, F8, F11, F12, F15, F9, F17, F18, F10, F20, F21, F22, F25, F27, F29, F30, F31, F32, F33, F34, F35, F38, F40	27	98.88%
	**3**	**F1, F4, F5, F6, F7, F8, F11, F12, F15, F17, F20, F21, F22, F23, F29, F30, F31, F33, F35, F39, F40, F41**	**22**	**99.13%**
	4	F1, F2, F8, F11, F12, F13, F15, F17, F19, F20, F21, F23, F24, F27, F29, F31, F32, F35, F38, F40, F41, F42	22	98.81%
	5	F1, F2, F7, F11, F12, F13, F14, F15, F17, F18, F20, F21, F23, F24, F26, F27, F28, F29, F31, F34, F37, F42	22	99.02%
ACO	1	F2, F3, F4, F5, F6, F7, F8, F9, F10, F11, F12, F13, F14, F15, F16, F17, F18, F19, F20, F21, F22, F23, F24, F25, F26, F27, F28	27	98.57%
	2	F1, F2, F3, F4, F5, F6, F10, F12, F13, F14, F15, F16, F17, F18, F19, F20, F21, F22, F24, F25, F26, F27, F29, F30, F31, F32, F33, F35, F36, F37, F38, F39, F40, F41, F42	35	98.74%
	3	F1, F2, F3, F4, F5, F6, F7, F9, F10, F11, F12, F13, F14, F15, F18, F20, F21, F22, F23, F24, F25, F27, F28, F30, F31, F32, F34, F37, F42	29	98.12%
	4	F1, F8, F13, F15, F16, F18, F19, F20, F21, F25, F31, F36, F37, F38, F39, F40, F4, F11, F14, F9, F17, F5, F7, F34	24	98.88%
	5	F1, F3, F4, F6, F8, F10, F11, F13, F16, F15, F18, F22, F23, F24, F26, F27, F28, F30, F31, F32, F33, F34, F35, F37, F41, F42	26	97.66%
PSO	1	F1, F2, F4, F5, F6, F8, F10, F11, F12, F13, F14, F17, F19, F20, F21, F23, F25, F26, F29, F33, F34, F35, F36, F39, F42	25	98.25%
	2	F1, F2, F4, F6, F8, F11, F12, F13, F14, F15, F17, F19, F20, F22, F23, F25, F26, F30, F31, F34, F35, F37, F38, F40, F41, F42	26	98.64%
	3	F1, F3, F5, F8, F9, F10, F14, F15, F16, F18, F20, F23, F24, F27, F28, F29, F30, F31, F33, F34, F35, F36, F37, F38, F40	25	98.95%
	4	F1, F2, F3, F5, F7, F10, F12, F13, F14, F16, F18, F20, F22, F26, F27, F28, F29, F30, F31, F33, F35, F37, F39, F42	24	97.87%
	5	F1, F2, F4, F6, F7, F10, F12, F13, F15, F16, F17, F18, F20, F21, F25, F27, F29, F32, F34, F36, F37, F40	22	98.88%

Bold group indicates the smallest number of features and has the highest classification accuracy.

**Figure 6:**
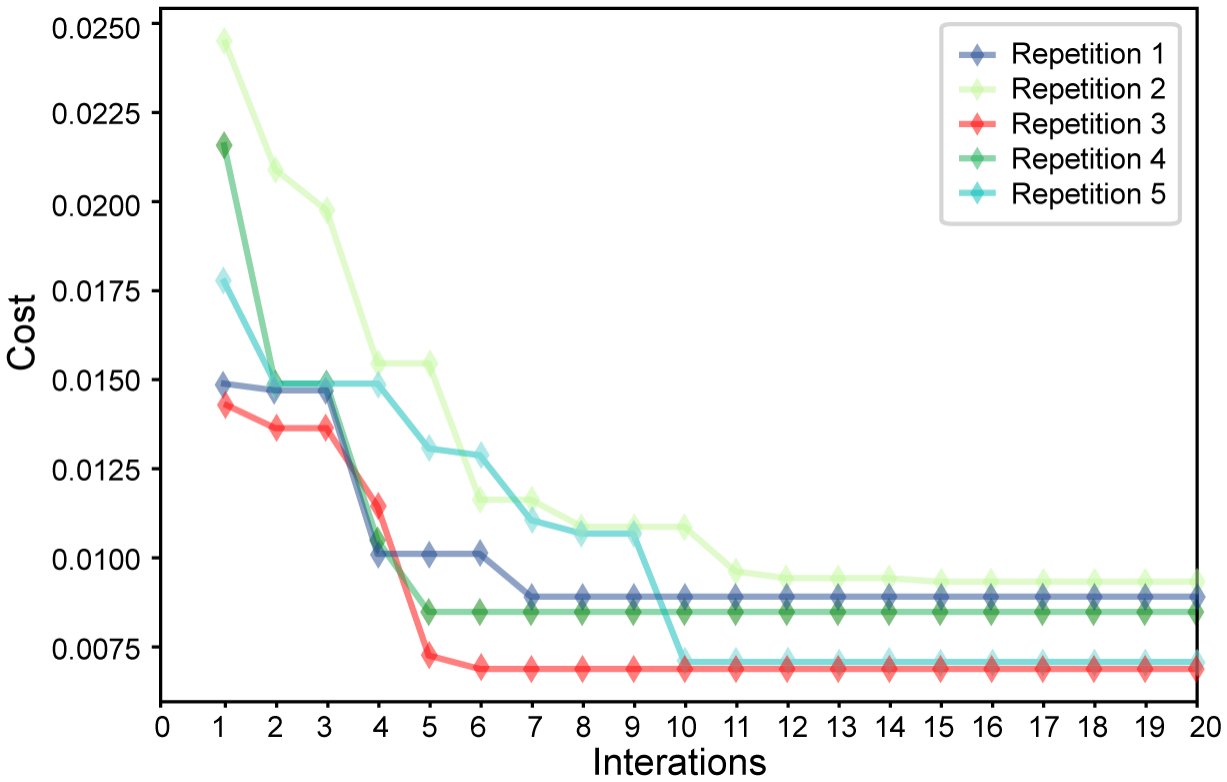
Variation in the evaluation function of five groups of feature sets.

Since the GA selects the optimal feature set, the change trend of the evaluation function (cost) based on the number of iterations for the five feature sets selected by the GA algorithm was calculated (**Figure 6**). The value of the evaluation function differs because the error rate and number of selection features constantly change during the iterative process. In this search process, since the GA is updated based on the outstanding individuals in the previous generation population, each generation population will retain at least the outstanding individuals of the previous generation. This feature allows the value of the evaluation function to be maintained and decreased. As a better feature set is found, the value of the evaluation function gradually decreases and finally converges to a stable point. This demonstrates that the algorithm successfully reduces the error rate and effectively reduces the length of the feature subset by identifying powerful features and ignoring redundant features. The evaluation function curve of the third group sharply decreases, converges in the shortest time, and determines the feature set with the lowest cost. Among the selected core features, 9 of the 14 newly proposed features were selected (namely, F4, F5, F6, F7, F8, F15, F22, F23, and F31), indicating that our newly proposed features play an important role in the classification of wintersweet.

### 3.4 Correlation analysis of all features and core features

**Figure 7:**
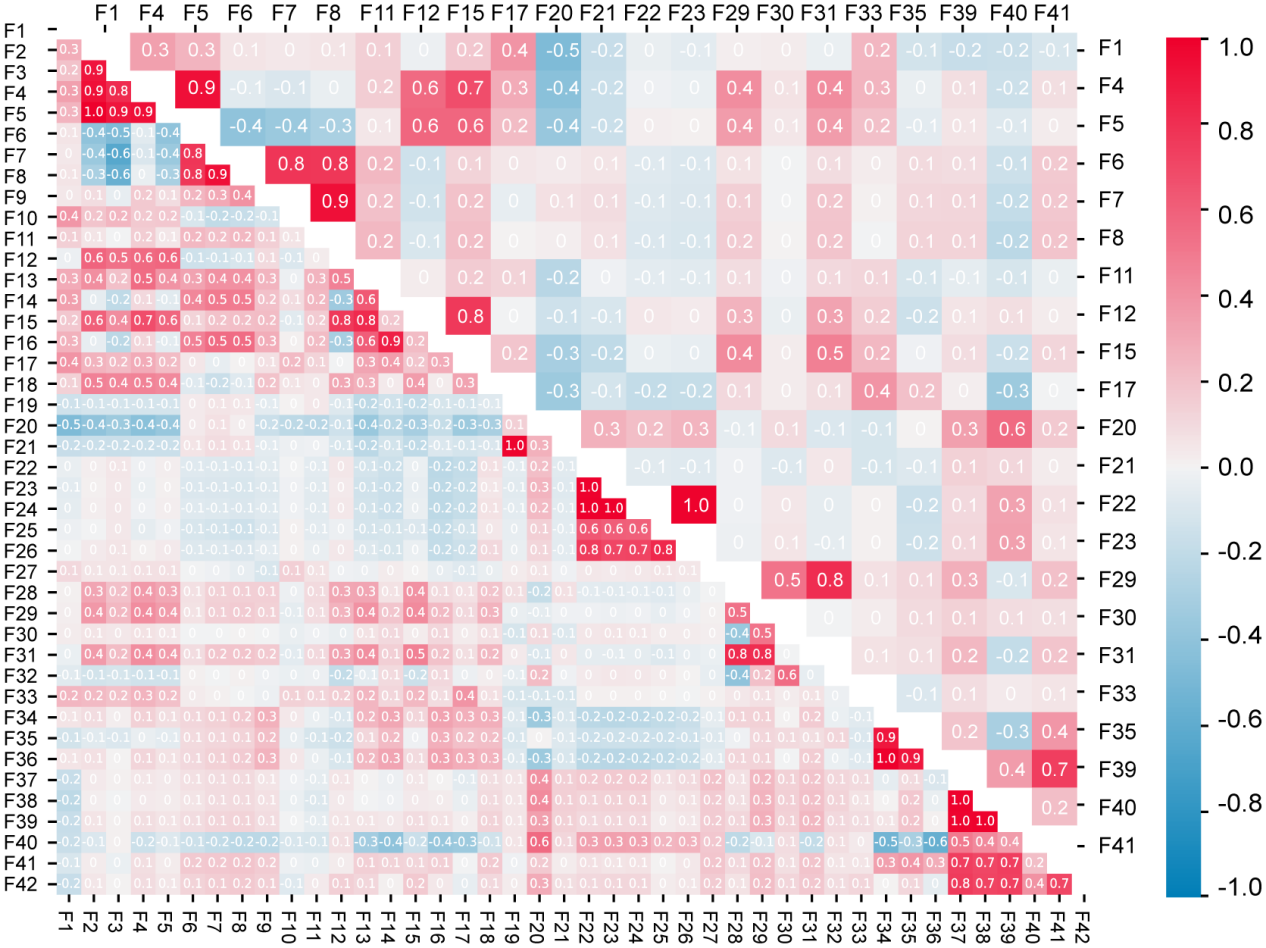
Correlations between all the traits (lower triangle) and the core traits (upper triangle).

Correlation analysis was performed using all 42 features and 22 core features respectively, as shown in **Figure 7**. According to the correlation analysis results for all the features, strong correlations (Pearson correlation coefficient | *r* | ≥ 0.5) were found among F2, F3, F4, F5, and F12; F6, F7, F8, and F16; F12, F13, and F15; F22, F23, F24, F25, and F26; F28, F29, and F31; F34, F35, and F36; and F37, F38, F39, F41, and F42. Among the 42 features, many had a high correlation. Moreover, the variance inflation factor (VIF) of most features was greater than 10 (**Supplementary Table S1**). This indicates the existence of a multicollinearity problem between the features. The above calculations and analyses show that information redundancy exists among these features.

The correlation analysis of the 22 core features after selection found that some features of the above highly correlated features were deleted. Similarly, the VIF was calculated for the 22 core features (**Supplementary Table S1**). The results showed that the VIFs of all the features significantly decreased, and the core features alleviated the influence of multicollinearity on the classification model. The above results indicate that the core feature set selected by the GA reduces redundancy among features, thereby improving the classification efficiency of wintersweet varieties.

### 3.5 Model performance and t-SNE visualization of the datasets

**Figure 8:**
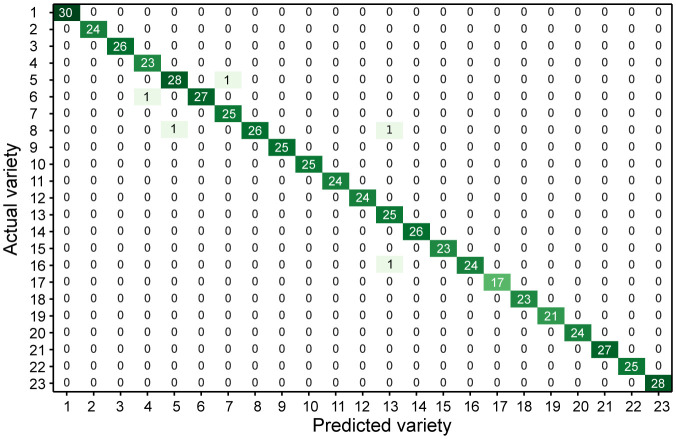
Core feature set using the confusion matrix of the RF model.

The core feature set was used to identify varieties and construct a confusion matrix (**Figure 8**). A confusion matrix was used to evaluate the performance of the classification model, and the relationships between different varieties were also visually observed. **Figure 8** shows the performance results on a divided test set with a total of 2,300 data samples. The overall recognition accuracy was 99.13%. Thus, the core feature set obtained by feature selection could be used to effectively identify wintersweet cultivars via the RF model, thus demonstrating the effectiveness of selecting the core feature set based on the GA for wintersweet cultivar classification. This strategy has the advantage of reducing feature dimensions and better utilizing the effective search space. However, some varieties were not well identified; for example, varieties 5, 6, 8, and 16 were identified as varieties 7, 4, 5/13, and 13, respectively, because these varieties are similar in appearance and can be easily confused with each other.

**Figure 9:**
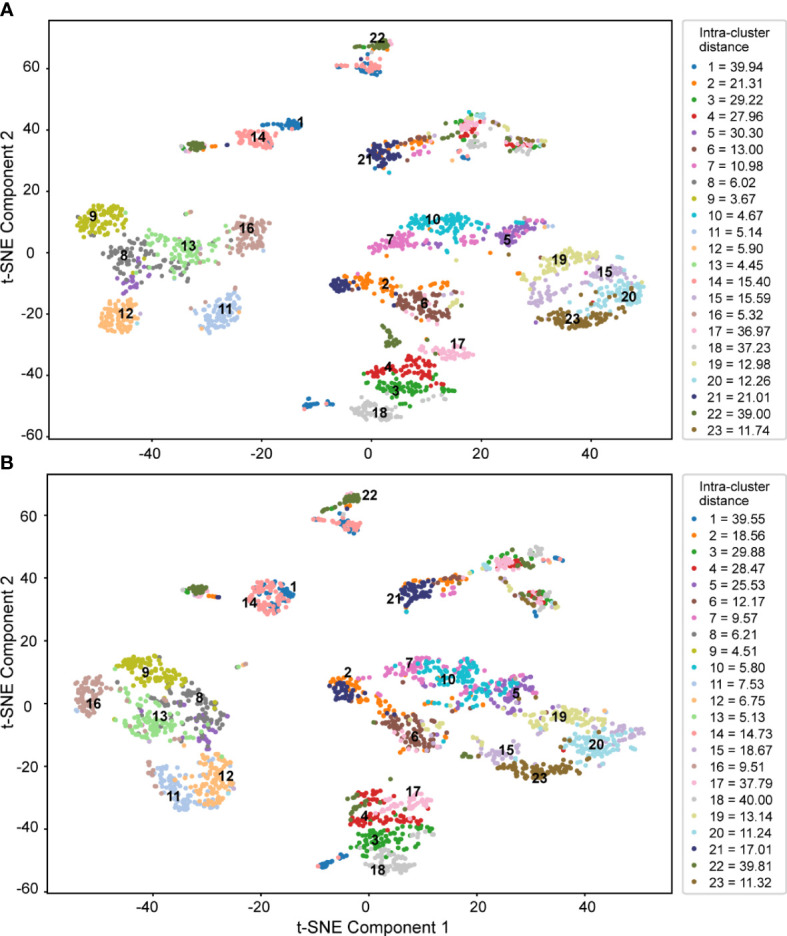
T-SNE dimensionality reduction of **(A)** all features and **(B)** core features.

Both sets of features, all features (**Figure 9A**) and core features (**Figure 9B**), were considered for t-SNE dimensionality reduction. The image features of 2,300 wintersweet flowers were visualized in the first two projection spaces, and different colors were selected based on the variety to directly observe the feature distribution of the images of different varieties extracted by the model. The two scatter plots show that the first two projection spaces have a high explanatory ability for evaluating the image features of the 23 varieties of wintersweet. After dimensionality reduction using different numbers of features, the distribution results for the 23 varieties were essentially the same. Moreover, overall, the intra-cluster distance was small (right side of the figure), whereas the inter-cluster distance was large (**Supplementary Table S2**), thus indicating that the varieties could be clearly distinguished. This further proves that the core feature set has a strong ability to accurately distinguish between different species of wintersweet.

As shown in **Figure 9**, the samples of varieties 8, 9, 10, 11, 12, 13, and 16 were closely aggregated, with a small intra-cluster distance and good classification effect. Varieties 1, 2, 17, and 22 exhibited poor aggregation and relatively large intra-cluster distances. Additionally, some varieties overlapped; these included 1 and 14, 2 and 21, 13 and 8, 8 and 5, and 5 and 7. The flowers of these overlapping varieties had similar colors or shapes. Furthermore, the varieties that were easily misclassified in the confusion matrix and some varieties that overlapped in the t-SNE dimensionality reduction were the same, such as varieties 5 and 7, 8 and 5, and 8 and 13, because these varieties were closely related. Therefore, all the varieties were clustered to explore their genetic relationships.

### 3.6 K-means clustering of 23 varieties

**Figure 10:**
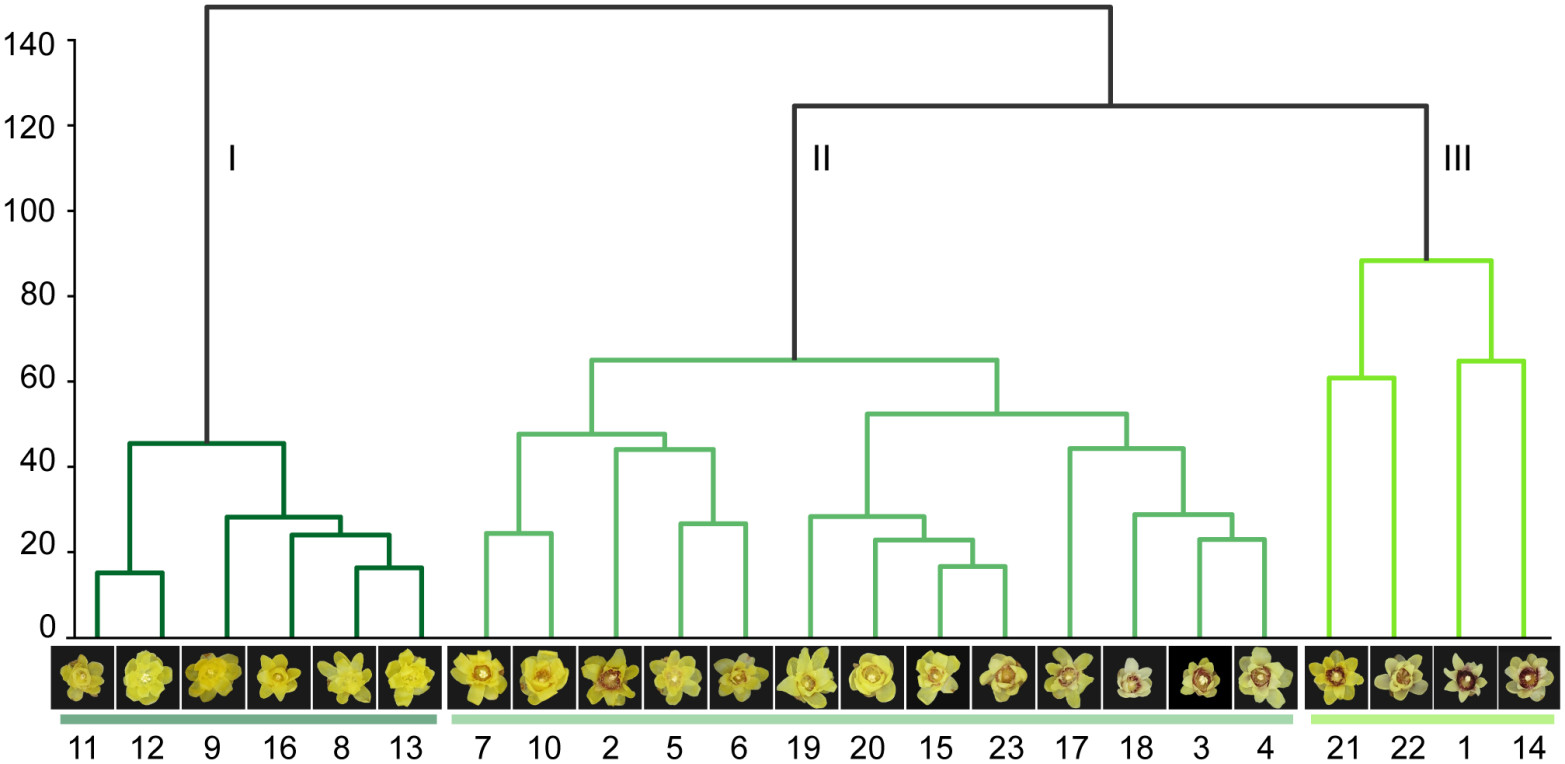
Twenty-three varieties of cluster trees.

K-means clustering was performed using the average value of each variety feature to explore the relationships between the varieties (**Figure 10**). According to the classification system proposed by (Chen, 2012), wintersweet can be divided into three groups: the concolor, intermedius, and patens groups. The clustering results of this study showed that the 23 varieties could be divided into three groups. Varieties 11, 12, 9, 16, 8, and 13 were clustered in group I. These varieties belonged to the concolor group; that is, their inner perianth is yellow without purple. In the other two groups, II and III, the inner perianth was purple. Among them, most varieties in group II belonged to the intermedius group, whose inner perianth was covered by a small amount of purple (red) color, whereas group III primarily belonged to the patens group, whose inner perianth was purple (red) in color. This finding highlights the importance of flower color in the classification of wintersweet. The k-means clustering results showed that the misclassified varieties in the confusion matrix were clustered into one group. Similarly, in the t-SNE dimensionality reduction visualization, varieties prone to overlap were clustered more closely. These results prove that these varieties are closely related.

## 4 Discussion

### 4.1 Evaluation of variety recognition ability

Recently, the rapid development of computer science has powered research and development in various disciplines. Remarkable progress has been made in the field of plant recognition based on machine learning (Sachar and Kumar, 2021; Wang et al., 2023), which has promoted the interdisciplinary development of plant research. However, research combined with DUS testing is currently limited.

In this study, a pattern recognition-related method was first used to efficiently quantify the shape, size, color, and other features of wintersweet. Compared with traditional manual measurements, image analysis provides more objective quantitative information; thus, it can rapidly and accurately process a large amount of data and improve the efficiency of identification. Herein, features including the description of flower traits in the DUS test and some newly proposed traits, totaling 42 features, were considered and classified. Eight algorithms were used to classify the varieties based on different feature sets. Overall, the RF algorithm exhibited the best performance. Based on the feature set in the DUS test, the identification accuracy of the varieties reached 98.43%, and it had a strong ability to distinguish them, thus verifying the effectiveness of the DUS test guidelines. Similarly, in the identification of peanut varieties, five form features were used, and the DUS test traits had the best recognition abilities (Deng and Han, 2019). The DUS test plays an important role in the identification and protection of new plant varieties. Therefore, the traits mentioned in the DUS test have a strong ability to be recognized. In this study, the recognition accuracy improved when the new features were used in the classification, together with the traits in the DUS test, thus indicating that the new features contribute to the classification of wintersweet varieties.

When the features of different parts were used to distinguish between the varieties of wintersweet, the differentiation ability of the outer perianth was the strongest, followed by that of the inner perianth. In previous studies on the classification of wintersweet, many researchers believed that the relevant features of the outer perianth should be used as the first classification standard. For example, Zhao et al. (2004) used clustering and PCA to determine that the length of the outer perianth should be used as the first classification standard. The results of Jing (2008) and Zhang (2010) proved that the length-width ratio (shape) of the outer perianth plays the most important role in clustering. Subsequently, Cheng and Zhou (2012) reported that the color of the outer perianth should be used as the primary standard for classifying varieties. These conclusions were drawn possibly because the outer perianth contains a wealth of information. First, the color information of the outer perianth is crucial, the color of the upper petal represents the color of the entire flower, whereas the purple area and color value at the bottom of the petal reflect various other information in the patens group. Moreover, the length of the outer perianth reflects the size of the flower, and the shape of the outer perianth may be related to the overall shape of the flower.

Among the eight algorithms, in the ranking of the first 15 important features of each algorithm, the features with the highest importance and more occurrence times were almost all related to the traits of the outer perianth and the color traits of the inner perianth. Zhao et al. (2007); Sun (2007); Lu and Sun (2008), and Ren (2010) believed that the color of the inner perianth is a crucial standard for the classification of wintersweet. Based on the results of this study and those of previous studies, the color, shape, size, and other features of the outer perianth and the color features of the inner perianth play important roles in the classification of wintersweet.

### 4.2 Improvement in identification efficiency using core features

An efficient classification model should obtain the maximum amount of information using the smallest number of features. A large number of features can lead to redundancy and noise, increase the computational burden, and reduce the classification effect (Ghasab et al., 2015; Khan et al., 2019). In previous studies on the classification of wintersweet varieties (Zhao et al., 2004; Zhao et al., 2007; Lu and Sun, 2008), researchers primarily employed PCA to reduce the dimensions of the characteristics and classified them by obtaining comprehensive indicators with nonoverlapping information. In terms of feature processing, PCA is computationally simple; however, it may ignore important features related to the target variables. In this study, a GA based on the wrapper strategy was used to search the core feature set for wintersweet identification. This method can more comprehensively search the feature subset space and determine near-optimal feature combinations. Of the total 42 features, from the DUS test and newly proposed features, 22 core features were selected using the feature selection method proposed in this experiment, and the recognition accuracy improved from 98.81% to 99.13%. After the selection, the number of features was reduced by approximately half, and the recognition accuracy was significantly improved. This feature combination can more accurately identify wintersweet varieties. In a previous study on apple disease identification and recognition methods, a GA was used to select the best features, and multi-SVM was used for classification. The results showed that the proposed feature selection method performed well in terms of both accuracy and execution time (Khan et al., 2019).

Correlation analysis of all features and core features revealed that some features had a high correlation among all features, and some of them were eliminated in the core feature set. This indicates that the 22 features of the core feature set contain more key attributes that can accurately describe the classification of wintersweet varieties and can thus reduce redundant information, improve the accuracy and efficiency of wintersweet classification, and better meet the needs of classification tasks.

### 4.3 Reliability of phenotypic characteristics for the identification of wintersweets

The core features were used for confusion matrix analysis for evaluating and verifying the performance of the classification model for wintersweet variety identification. The overall recognition accuracy of the confusion matrix calculation was high. However, some varieties were prone to misclassification. t-SNE was used to reduce the dimensionality of all the features and the core features. After dimensionality reduction, the aggregation results of the two feature sets for the different varieties were basically the same, thus demonstrating the strong explanatory power of the core features. The genetic relationships of the 23 varieties were analyzed using k-means clustering, and according to the results, the 23 varieties were grouped into three groups. Group I was the concolor group, whereas the inner perianth of the other two groups was purple. Interestingly, in the cluster analysis, the varieties misclassified by the confusion matrix and the varieties overlapping in the t-SNE dimensionality reduction showed relatively close relationships. Note that the use of phenotypes for variety identification and phylogenetic analysis has been previously confirmed for several plants. Lu and Du (2007) used flower morphology and RAPD markers to classify the varieties of wintersweet, and the results were consistent. Jing (2008) showed that flower morphology and ISSR markers were consistent in judging the morphological evolution and genetic relationships of wintersweet plants. This finding demonstrated the reliability of phenotypic characteristics in identifying wintersweet varieties. Similar results have been reported for other species as well. During cucumber variety identification, the DUS and SNP markers exhibited highly similar variation curves, thus confirming the consistency of the DUS and DNA fingerprinting results for cucumber variety identification (Zhang et al., 2022). Deng and Han (2019) used 18 phenotypic features to cluster 20 peanut varieties, and the clustering results were significantly affected by pod type and origin. Morphological data play an indispensable role in investigating maize polymorphisms and genetic relationships. Only by utilizing morphological data as auxiliary information for molecular markers can effective distinction maize strains and conduct genetic relationship analysis be achieved (Nagy et al., 2003). Therefore, identifying phenotypic traits is regarded as an effective method for identifying plant varieties.

In future research, we will collect new wintersweet flower image data and use the 22 core features of this study for classification to verify the validity and generalizability of the feature set. Machine learning-based feature selection methods have considerable potential for application in plant variety identification. Our study provides a novel idea for future DUS testing of wintersweet. Additionally, these findings can guide and support plant variety classification, protection, and breeding.

## Data availability statement

Pseudocode for GA can be found on GitHub (https://github.com/csparow/GA). Other raw data supporting the conclusions of this article will be made available by the authors, without undue reservation.

## Author contributions

TZ: Conceptualization, Data curation, Writing – original draft, Writing – review & editing. YF: Formal analysis, Methodology, Software, Writing – original draft. XD: Methodology, Software, Writing – original draft. XY: Writing – review & editing. BL: Data curation, Writing – review & editing. PY: Data curation, Writing – review & editing. XS: Data curation, Writing – review & editing. SC: Supervision, Writing – review & editing. SS: Conceptualization, Funding acquisition, Supervision, Writing – review & editing.
